# Urinary Nitric Oxide Levels Are Associated with Blood Pressure, Fruit and Vegetable Intake and Total Polyphenol Excretion in Adolescents from the SI! Program

**DOI:** 10.3390/antiox11112140

**Published:** 2022-10-28

**Authors:** Sonia L. Ramírez-Garza, Emily P. Laveriano-Santos, Camila Arancibia-Riveros, Jose C. Carrasco-Jimenez, Patricia Bodega, Amaya de Cos-Gandoy, Mercedes de Miguel, Gloria Santos-Beneit, Juan Miguel Fernández-Alvira, Rodrigo Fernández-Jiménez, Jesús Martínez-Gómez, Ramón Estruch, Rosa M. Lamuela-Raventós, Anna Tresserra-Rimbau

**Affiliations:** 1 Departament de Nutrició, Ciències de l’Alimentació i Gastronomia, Xarxa d’Innovació Alimentària (XIA), Facultat de Farmàcia i Ciències de l’Alimentació, Institut de Nutrició i Seguretat Alimentària (INSA-UB), Universitat de Barcelona (UB), 08028 Barcelona, Spain; 2 Consorcio CIBER, M.P. Fisiopatología de la Obesidad y Nutrición (CIBERObn), Instituto de Salud Carlos III (ISCIII), 28220 Madrid, Spain; 3 Barcelona Supercomputing Center (BSC), 08034 Barcelona, Spain; 4 Foundation for Science, Health and Education (SHE), 08008 Barcelona, Spain; 5 Centro Nacional de Investigaciones Cardiovasculares (CNIC), 28029 Madrid, Spain; 6 The Zena and Michael A. Wiener Cardiovascular Institute, Icahn School of Medicine at Mount Sinai, New York, NY 10029, USA; 7 Hospital Universitario Clinico San Carlos, 28040 Madrid, Spain; 8 Centro de Investigación Biomédica en Red en Enfermedades CardioVasculares (CIBERCV), 28029 Madrid, Spain; 9 Department of Internal Medicine, Hospital Clínic, Institut d’Investigacions Biomèdiques August Pi i Sunyer (IDIBAPS), Universitat de Barcelona, 08036 Barcelona, Spain

**Keywords:** antioxidant, teenager, adolescents, diet, cardiovascular health, ideal cardiovascular health, ICH

## Abstract

Nitric oxide (NO) is important to cardiovascular health (CVH), and its bioavailability could be regulated by the antioxidant effect of polyphenols, improving endothelial function and consequently blood pressure (BP). However, scant research has been carried out on NO and CVH correlates in adolescent populations. Therefore, our aim was to investigate the association between NO and the CVH status and other health factors in adolescents. NO, total polyphenol excretion (TPE), anthropometric measurements, BP, blood lipid profile, blood glucose, diet, physical activity, and smoking status were recorded, while CVH score was classified as ideal, intermediate, and poor. Negative associations were observed between NO and body mass index, body fat percentage, BP, and triglycerides; and positive associations between NO and skeletal muscle percentage, HDL-cholesterol, fruit and vegetable intake, and TPE was observed. To capture more complex interactions among different factors, multiple linear regression was performed, obtaining a significant association between NO and fruit and vegetable intake (β = 0.175), TPE (β = 0.225), and systolic BP (β = −0.235). We conclude that urinary NO levels are positively associated with the consumption of fruits and vegetables rich in antioxidants such as polyphenols and negatively associated with systolic BP.

## 1. Introduction

Approximately one third of global deaths in 2019 were caused by cardiovascular diseases [1]. An increased cardiovascular risk is associated with the dysregulation of nitric oxide (NO) since it plays an important role in vascular homeostasis, which contributes to control blood pressure (BP) [2,3]. As a short-lived reactive gas, NO easily oxidizes to more stable nitrite and nitrate forms [4], and the excess is excreted by urine (around 75%) and saliva [5]. NO can be produced endogenously by the L-arginine-NO synthase pathway or by the reduction of dietary nitrates and nitrites found in vegetables, fruits, and some meat products [2,6]. In fact, approximately 80% of nitrate intake comes from vegetables, mainly beets and green leaves such as raw spinach, rucola, celery, etc. [5,7,8], and the best sources found in fruits include melon, strawberries, and banana [7].

In addition, fruits and vegetables are sources of antioxidants such as polyphenols, which could improve the bioavailability of NO [9,10]. As a consequence, a diet rich in fruits and vegetables could result in an increase of nitrate, nitrite, and antioxidant, which could contribute to improve cardiovascular health [7,11,12].

Some studies have evaluated the relationship between NO and nitrate intake in adult populations, showing a reduction in blood pressure after a dose of nitrate whether in concentrated solutions or in the form of vegetable-based products [2,8]. However, scarce studies have examined the association between a healthy diet (i.e., fruit and vegetable intake) and urinary NO.

On the other hand, tobacco contributes to a decrease in the activity of NO synthase (neuronal and endothelial), which decreases NO production in smokers [13,14]. Moreover, inconsistent results were observed in the relationship between NO and body mass index (BMI) since positive associations have been observed in children [15], while another study reports no association in adolescents [16]. In addition, NO plays an important role in endothelial function [17], producing an antihypertensive effect, owing to blood vessel dilation due to a relaxation of vascular smooth muscle, thus reducing blood pressure [3,18]. However, diverging results have been reported in the relationship between NO and BP [2,16,19,20,21,22,23].

The American Heart Association defines cardiovascular health (CVH) as the presence of four health behaviors: smoking status, BMI, physical activity, and diet status, and three health factors: blood glucose levels, total cholesterol (TC), and BP [24]. Due to the scant research on the relationship between CVH components and NO in adolescents, we ask ourselves whether the habits of our adolescents, without previous diagnoses of cardiovascular pathologies, have any relationship with cardiovascular factors? Is the relationship between NO and CVH similar for adolescents as in adult populations? Therefore, the aim of the present study was to examine the association between NO and CVH status and other health factors in a cohort of adolescents enrolled in the SI! Program for Secondary Schools in Spain.

## 2. Materials and Methods

In this section, we describe the study design, sample selection, anthropometric, and cardiovascular measurements, urinary biochemical and statistical analyses. Figure S1 depicts a schematic view of the process of the different phases of the present study.

### 2.1. Study Design and Sample Selection

A cross-sectional analysis of baseline data was performed in a sub-sample of participants enrolled in the SI! Program for Secondary Schools trial (registered at ClinicalTrials.gov: NCT03504059, accessed on 3 October 2022). This study enrolled a total of 1326 adolescents from 24 public secondary schools in Spain (seven in Madrid and 17 in Barcelona). All participants signed an informed consent form. All the details of the recruitment and design of the trial have been previously published [25]. For the purpose of the present sub-study, a minimum of 147 participants were required to provide 90% power of test and a significance level of 5% when performing multiple regression. However, we analyzed 150 urine samples, one of which was lost in the process. Participants enrolled in the SI! Program at 1st grade of secondary school was the inclusion criterion (aged 12 years); and exclusion criteria included: no fasting, no urine sample, or missing measurements of cardiovascular health score. A simple randomization strategy was used to select a sample of participants (more details in flow chart in Figure S2). The Kolmogorov–Smirnov test was used to ascertain that study variables (except NO) exhibited no significant difference between the selected sample and the overall population included in the SI! Program for Secondary Schools trial.

### 2.2. Anthropometric Measurements

All anthropometric measurements were performed by trained nutritionists. Height was measured with a Seca 213 portable stadiometer (0.1 cm of precision). Body weight, body fat percentage, and skeletal muscle percentage were measured using an OMRON BF511 electronic scale with 0.1 Kg of precision (OMRON HEALTHCARE Co., Muko, Kyoto, Japan) after overnight fasting, with participants wearing light clothes and no shoes. BMI was calculated as body weight divided by height squared (kg/m^2^). Waist circumference was measured three times with a Holtain tape to the nearest 0.1 cm [25]. Waist-to-height ratio was calculated as waist circumference divided by height [26]. BMI, waist circumference, and waist-to-height ratio were adjusted by age and sex to obtain z-scores values according to Cole and Lobstein, 2012 and Sharma et al., 2015, respectively [27,28].

### 2.3. Cardiovascular Measurements

Smoking status was assessed by answering a questionnaire [29] in which the participants reported whether they ever smoked tobacco products (e.g., cigarettes, electronic cigarettes, or hookah). Moderate and vigorous physical activity levels were estimated by the triaxial accelerometer (Actigraph wGT3X-BT, ActiGraph Corporation, Pensacola, FL, USA) worn on the non-dominant wrist for 7 consecutive days. Activity information was considered valid if data were available from a minimum of 4 days with at least 600 min of wear time per day. Physical activity intensities were estimated using the cut-off points of Chandler et al., 2016 [30]. BMI percentiles were calculated according to Centers for Disease Control standards [31]. BP was measured with an OMRON M6 monitor (OMRON HEALTHCARE Co., Muko, Kyoto, Japan) with 2–3 min intervals between measurements. When the differences between the measurements were less than 10 mmHg for systolic blood pressure (SBP) and less than 5 mmHg for diastolic blood pressure (DBP), two measurements were taken. Otherwise, a third reading was performed [25]. Average values were calculated for the final SBP and DBP, which were adjusted by age, sex, and height to obtain percentile and z-score values [32]. Biochemical blood analysis was performed by trained nurses using samples taken early in the morning after overnight fasting. Glucose, triglycerides, TC, high density lipoprotein cholesterol, low density lipoprotein cholesterol, and non-high density lipoprotein cholesterol were determined using the Cardio Check Plus device and PTS Panels test strips for capillary blood samples [33]. Dietary data and information about pathologies or food allergy and intolerance (Table S1) were obtained using a validated food frequency questionnaire filled out by participants [34,35], and another validated 157-item food frequency questionnaire filled out by the families of the participants to provide complementary dietary information [36], which was used, along with the food composition tables of CESNID [37], to estimate intake of sodium, vitamin A, carotenoids, retinol, vitamin E, and vitamin C. The healthy diet score was assessed by five components: fruits and vegetables ≥ 4.5 servings/day, fish ≥ 2 servings/week, fiber-rich whole grains ≥ 3 servings/day, and sugar-sweetened beverages ≤ 1 L/week and sodium ≤ 1500 mg/day. Classifications of healthy diet scores were 4–5 components for ideal, 2–3 components for intermediate, and 0–1 components for poor diet [24].

CVH score (CVHs) was calculated based on the 7 cardiovascular metrics (i.e., smoking status, moderate and vigorous physical activity, BMI, BP, blood glucose, TC, and diet) using the cut-off points proposed by Steinberger et al., 2016 [38]. Each component was then dichotomized as ideal (1 point) versus non-ideal (0 points) and scored from 0 to 7 points, with a higher score indicating a better CVHs profile. Classification categories were 0–3 for poor, 4–5 for intermediate, and 6–7 for ideal [39,40].

### 2.4. Urinary Biochemical Analyses

All participants in this study provided a fasting spot urine sample, collected in the morning. The urine was aliquoted and stored at −80 °C for subsequent analysis [25]. NO concentration in urine was determined by the Griess method using the Nitrate/Nitrite Colorimetric Assay Kit (Cayman Chemical, Ann Arbor, MI, USA, Item No. 780001). Following instructions of the protocol, enzymatic reductase converts nitrate to nitrite. Then, Griess reagent converts the nitrite into a deep purple azo compound. Values obtained from the Griess assay represent the sum of nitrite and nitrate (NO). Creatinine was measured by the validated Jaffé alkaline picrate method and total polyphenol excretion (TPE) by Folin-Ciocalteu spectrophotometric methods [41]. NO and TPE levels were normalized by creatinine and the results were expressed as μM NO/mM creatinine and mg gallic acid equivalent/g creatinine (respectively).

### 2.5. Statistical Analyses

A six-step process was followed to identify the association among NO and CVH status and other healthy factors. First, to prevent variables from having unequal weights, all variables were standardized (z-scores) prior to the statistical analysis. Second, the Kolmogorov–Smirnov test was used to determine normality of variables. Third, candidate predictor variables were identified using a variety of statistical methods: Student *t* test was used to identify variables that exhibited sex-related differences: Kruskal–Wallis test with Dunn–Bonferroni correction was used to ascertain for differences between CVHs categories, and to identify differences in NO levels among CVHs, and eta-squared was used to identify effect size; correlation between NO levels and each study variable was assessed with simple linear regression and Spearman’s correlation coefficients. Fourth, principal component analysis was used to eliminate collinearity among variables. Fifth, to identify the most important variables associated with NO, the forward stepwise selection method was used. This method was fed with the statistically significant variables (with *p*-values < 0.05) from the analysis of differences between sexes (Student *t* test), variables with different median values in the CVHs categories (Kruskal–Wallis tests), and correlated variables (simple linear regression analysis and Spearman’s test) with NO. Finally, with the subset of variables selected by the forward stepwise method, a multiple linear regression model, adjusted by confounding factors, such as sex, age, smoking status, and BMI, was used to determine the influence and global association of study variables with NO additionally. Data were analyzed with R software version 4.1.0 (R Studio, 250 Northern Ave, Boston, MA, USA). In all tests, a *p*-value < 0.05 was considered statistically significant.

## 3. Results

The general characteristics of the study population are presented in Table 1. A total of 149 adolescents, 49.0% boys and 51.0% girls with a median age of 12.0 ± 0.5 years, were included. Girls had lower values for waist-to-height ratio z-score, skeletal muscle percentage, and SBP z-score, but higher for body fat mass percentage.

Table 2 shows significant differences among CVHs categories: in moderate and vigorous physical activity between the ideal and intermediate categories; in BMI, SBP, DBP, blood glucose, and TC between the poor category and the ideal and intermediate categories. No significant differences in NO or diet components were found among the CVHs categories (more details in Table S2). Three participants of the intermediate category and one participant from the poor category reported pathologies, respectively. Furthermore, six and four participants reported food allergy in the intermediate and poor categories. Lastly, one participant in each category group reported food intolerance (Table S3). A significant association was observed in simple linear regression between NO and BMI z-score, body fat percentage, skeletal muscle percentage, SBP z-score, DBP z-score, triglycerides, high-density lipoprotein cholesterol, TPE, and fruit and vegetable intake (Table 3).

Most of the variables correlated with NO levels according to the Spearman correlation test (Table 4) showed sex-related differences. Additionally, as indicated in Table 4, boys showed a significant inverse correlation between age and NO (Rho = −0.344), and a significant positive correlation between TPE (Rho = 0.375) and NO, whereas neither correlation was found in girls. On the contrary, girls showed a significant negative correlation between NO and BMI z-score (Rho = −0.261), body fat percentage (Rho = −0.232), DBP z-score (Rho = −0.317), and intake of whole grains and sugar-sweetened beverages (Rho = −0.349 and Rho = −0.296 respectively), whereas in boys these correlations were not statistically significant. Moreover, a significant positive correlation was found between NO and TC (Rho = 0.353), high-density lipoprotein cholesterol (Rho = 0.262), and low density lipoprotein cholesterol (Rho = 0.272) in girls, but not in boys. A significant negative correlation between NO and SBP z-score was observed in both sexes (Rho = −0.304 in boys and Rho = −0.391 in girls).

No collinearity was found among the variables, which was tested using principal component analysis. Skeletal muscle percentage, SBP z-score, TC, TPE, and intake of fruit and vegetables, whole grains, and sugar-sweetened beverages were identified by the forward stepwise method as the most important variables associated with NO.

**Figure 1:**
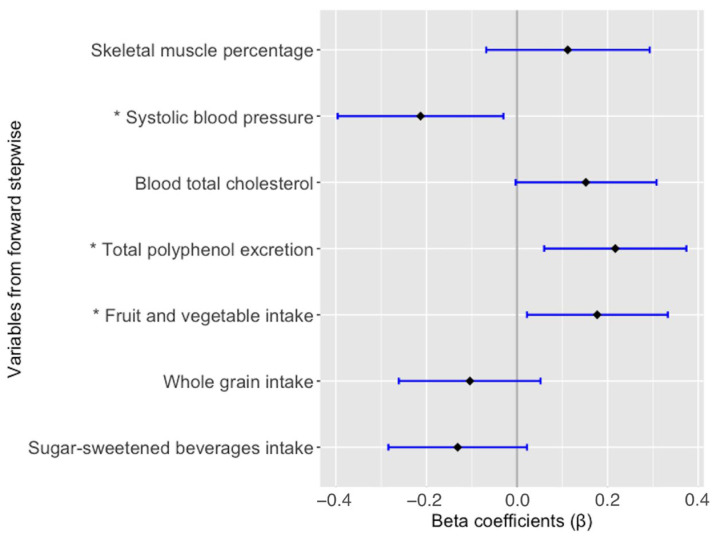
Multiple linear regression model for the association of nitric oxide. Adjusted by sex, age, smoking status, and BMI. All variables are expressed as z-score. * Statistically significant difference (*p*-value < 0.05).

Finally, fruit and vegetable intake (β = 0.175; *p* = 0.025), TPE (β = 0.225; *p* = 0.004) and SBP z-score (β = −0.235; *p* = 0.004) showed a statistically significant association with NO, whereas TC (β = 0.140; *p* = 0.071), skeletal muscle percentage (β = 0.111; *p* = 0.190), and the intake of sugar-sweetened beverages and whole grains (β = −0.131; *p* = 0.091 and β = −0.104; *p* = 0.189, respectively) were not significantly associated with NO in the multiple linear regression model adjusted by confounding factors (Figure 1).

## 4. Discussion

In this cross-sectional study performed in early adolescents enrolled in the SI! Program for Secondary Schools, NO levels were found to be positively associated with fruit and vegetable intake and TPE, and negatively associated with SBP z-score.

Similar findings have been observed in adult populations in a variety of studies assessing different combinations of the aforementioned factors. For example, a significantly increased plasma nitrate level was found in a healthy adult population after a one-week intake of approximately 340 mg of nitrate through beetroot juice, green leafy vegetable juice, or nitrate-rich vegetables. Moreover, in the same studies, a decrease of SBP and DBP was observed [42,43]. In the same line, plasma nitrate/nitrite levels increased significantly in overweight or obese adolescents after 1 h of intake and after a week of daily intake of 50 g of freeze-dried strawberry powder [44]. Likewise, a reduction in BP and an increase of NO were observed after the consumption of beetroot, spinach, or rocket leaf [2,8], all good sources of nitrates and nitrites, like many other fruits and vegetables [8,45,46]. Conflicting results were found in studies with postmenopausal women, or adults with prehypertension or hypertension, in which a daily intake of 150–400 mg nitrate over periods ranging from 10 days to 12 weeks increased NO (measured by nitrate in urine or plasma) but did not reduce SBP or DBP [47,48,49,50]. The consumption of fruits and vegetables, as a source of polyphenols, could induce NO production by increasing endothelial nitric oxide synthase (eNOS) activity and reducing oxidative stress and inflammation [51,52].

The present study shows a positive association between TPE and NO in adolescents. In accordance with this finding, in a PREDIMED trial sub-study with 200 men and women aged 55–80 years and at high cardiovascular risk, a high intake of polyphenols was found to increase NO in plasma [53]. The same effect was observed in a randomized, placebo-controlled, crossover trial study with 12 men and 11 women who ingested approximately 40 g/day of cookies made with either 20 g of roasted black soybean powder (containing approximately 20.0 mg of total polyphenols) or 20 g of flour (containing approximately 3.2 mg of total polyphenols) for four weeks, with the same period for washout between treatments. Plasma and urine NO and polyphenol concentration increased in the group consuming the black soybean cookies [54]. Comparable outcomes were observed in a crossover controlled interventional study with 22 healthy men, in which urine NO and polyphenol levels were higher in those following a healthy diet of polyphenol-rich food compared with a low antioxidant diet with less than two servings of fruit and vegetables per day and avoiding the intake of cocoa products, coffee, and tea. These results were observed after two weeks of the aforementioned diets and one week of washout between diets [55]. Similar findings were reported in another randomized, placebo-controlled, crossover trial in healthy adult men, in which oral administration of pure flavonoids appeared to improve NO status, measured by the circulating concentrations of NO products. Possible mechanisms underlying these effects are the inhibition of NADPH oxidases (NOX) and activation of eNOS [56]. NOX are found in endothelial cells, and can be in NOX1, NOX2, NOX3, and NOX4 isoforms; its main function is transferring an electron from NADPH to oxygen, with superoxide production. An increase in NOX expression has been related with the beginning of cardiovascular diseases [57]. NOX are the main source of the reactive oxygen (ROS), producing superoxide, as well as hydrogen peroxide. In oxidative stress conditions, eNOS may become dysfunctional and NOX could induce ROS production from uncoupled eNOS, thereby converting the eNOS to a superoxide producer, and as a consequence a reduction in NO production [58]. Moreover, the antioxidant effect of polyphenols could explain their role in the upregulation of NO production. Polyphenols, such as flavanols, flavonols, ferulic acid, resveratrol, and curcumin, could inhibit NOX expression and upregulate eNOS, increasing the production of nitric oxide (NO) and contributing to decrease blood pressure [59,60,61]. Additionally, some researchers suggest that polyphenols could improve the metabolism of NO through the L-arginine-NOS pathway, enhancing the activity of eNOS, which is activated via Pi3K/Akt [60,62,63,64]. In addition, reactive oxygen species can be inhibited by polyphenols, thereby reducing oxidative stress and the upregulation of inflammatory markers within the endothelium and upregulating NO synthesis [51].

A negative association between NO and SBP z-score was found in the present study. Similar to our results, an inverse association between NO and BP was reported in a study with 177 adult subjects without symptoms of coronary atherosclerosis and 457 patients suspected to have coronary heart disease, with or without coronary lesions, in which NO was analyzed in the serum [23]. Conversely, a positive correlation between SBP and serum NO was found in a study carried out with apparently healthy Saudi adolescents (245 boys and 495 girls, aged 10–17 years) [20]. Similarly, a study conducted with 103 boys and 135 girls aged 11 years, who were of normal weight, overweight, obese, normotensive, or hypertensive, showed a positive association between NO in plasma SBP and DBP [16]. Controversial results were observed in a study with 120 male adolescents aged 14–17 in which the highest NO levels were observed in the prehypertensive group, followed by the normal BP group [19]. It is possible that factors, such as diet, age, sex, and ethnic group, among others, may contribute to the variations in NO and BP levels of the aforementioned studies. Thus, the antioxidants found in fruits and vegetables, such as polyphenols, may exert antihypertensive effects by favoring the correct production and function of NO, preventing its transformation into nitrile peroxide [2,43,65,66].

This study assesses the relationship between NO and CVH status and other healthy factors in adolescents. To our knowledge, this is the first study that shows the association, as a whole, between NO, blood pressure, and fruit and vegetable intake in an adolescent population.

Among the strengths of the study, therefore, is its focus on an adolescent population. Additionally, a holistic approach has been taken, focusing on a range of factors potentially relevant for the production of NO, including diet, physical activity, and polyphenol biomarkers. Other strong points are the multicenter design and the use of a standardized protocol, which reduced the information bias.

The limitations of the study include the size of the study population, the fact that the accelerometer was not worn while practicing water activities or some sport that required its removal (e.g., judo, basketball, etc.), and the health status of the population, as due to their early stage of life, most participants were healthy. Due to age and ethical issues, it was not possible to obtain a blood sample, which would have allowed a more comprehensive knowledge of NO. The cross-sectional design was another limitation of our study.

The results of the present study are promising. However, a deeper understanding of the relationship between NO and CVH factors in early life stages is necessary. Our results may contribute to a better understanding of the relationship between NO and CVH factors in early life stages (i.e., adolescence). They may also contribute to health promotion (e.g., to promote fruit and vegetable intake) and the prevention of cardiovascular disease development in adolescents, thus improving quality of life in adulthood. However, deeper studies might be required to understand more complex interactions between the aforementioned factors.

## 5. Conclusions

In conclusion, our results show that NO is positively associated with fruit and vegetable intake and TPE and negatively associated with SBP. Our results, along with those exhibited in related research works, suggest that NO and polyphenol levels could come from the consumption of fruits and vegetables, and the combination of the effects of NO and polyphenols could produce a decrease in BP.

## Figures and Tables

**Table 1:** General characteristics of the participants analyzed by sex.

	Total (*n* = 149)	Boys (*n* = 73)	Girls (*n* = 76)	*p*-Value
Age (months)	151 ± 6	151 ± 7	151 ± 5	0.951
Smokers (%) *	10.7	9.6	11.8	
MVPA (min/day)	73.9 ± 22.1	71.7 ± 22.2	76.9 ± 21.7	0.088
Anthropometric				
BMI (kg/m^2^)	20.8 ± 4.3	20.8 ± 4.3	20.9 ± 4.4	0.849
BMI z-score	0.80 ± 1.05	0.87 ± 1.03	0.72 ± 1.08	0.393
WC (cm)	73.0 ± 11.7	73.6 ± 12.2	72.5 ± 11.3	0.568
WC z-score	0.45 ± 0.86	0.48 ± 0.88	0.42 ± 0.85	0.635
WHtR	0.47 ± 0.07	0.47 ± 0.07	0.46 ± 0.07	0.248
WHtR z-score	0.07 ± 0.98	0.24 ± 0.92	−0.10 ± 1.01	0.034
Body fat (%)	24.0 ± 8.5	22.4 ± 8.2	25.5 ± 8.5	0.028
Skeletal muscle (%)	34.2 ± 3.6	35.4 ± 3.2	33.0 ± 3.6	<0.001
SBP (mmHg)	113 ± 11	114 ± 10	111 ± 12	0.169
SBP z-score	0.60 ± 1.00	0.76 ± 0.90	0.42 ± 1.07	0.037
DBP (mmHg)	67 ± 9	67 ± 11	68 ± 8	0.683
DBP z-score	0.46 ± 0.88	0.47 ± 0.94	0.46 ± 0.82	0.934
Blood biochemistry				
Glucose (mg/dL)	99.5 ± 11.1	99.8 ± 11.3	99.2 ± 10.9	0.755
Triglycerides (mg/dL)	76.6 ± 29.9	76.5 ± 35.3	76.7 ± 23.7	0.973
TotalCholesterol (mg/dL)	151 ± 28	152 ± 31	150 ± 25	0.657
HDL-c (mg/dL)	61.7 ± 14.3	60.9 ± 14.3	62.4 ± 14.4	0.524
LDL-c (mg/dL)	74.0 ± 23.6	75.8 ± 25.2	72.3 ± 22.1	0.374
Non-HDL-c (mg/dL)	89.3 ± 25.2	91.1 ± 28.1	87.5 ± 22.1	0.393
Urine analysis				
NO (μM NO/mM creatinine)	40.9 ± 22.2	43.5 ± 24.0	38.4 ± 20.3	0.162
TPE (mg GAE/g creatinine)	137 ± 107	146 ± 118	127 ± 97	0.286
Diet				
Fruits & vegetables (servings/day)	2.68 ± 1.95	2.89 ± 1.94	2.47 ± 1.94	0.189
Whole grains (servings/day)	0.38 ± 0.53	0.35 ± 0.52	0.42 ± 0.54	0.442
Sodium (mg/day)	3340 ± 1042	3492 ± 1095	3180 ± 967	0.093
Fish (servings/week)	3.28 ± 2.47	3.23 ± 2.40	3.33 ± 2.56	0.813
Sugar-sweetened beverages (L/week)	1.38 ± 1.74	1.59 ± 1.92	1.19 ± 1.53	0.166
CVHs	4.27 ± 1.31	4.15 ± 1.32	4.38 ± 1.30	0.283

Values are expressed as mean ± SD. *p*-values are of *t*-test to refer differences between boys and girls. MVPA: Moderate & vigorous physical activity, BMI: body mass index, WC: waist circumference, WHtR: waist to height ratio, SBP: systolic blood pressure, DBP: diastolic blood pressure, HDL-c: high-density lipoprotein cholesterol, LDL-c: low-density lipoprotein cholesterol, NO: nitric oxide TPE: total polyphenol excretion, GAE: gallic acid equivalent, CVHs: cardiovascular health score. * Dichotomous variable is expressed as a percentage. A *p*-value < 0.05 was considered statistically significant.

**Table 2:** Categories of cardiovascular health score.

	Ideal (*n* = 28)	Intermediate (*n* = 77)	Poor (*n* = 44)	Effect Size	*p*-Value
Boys (%)	39.3	50.6	52.3		
Smokers (%) *	0.0	14.3	13.6		
MVPA (min/day)	81 (61–138) ^A^	71 (23–112) ^A^	74 (21–139)	0.034	0.030
BMI (percentile)	52.3 (6.7–78.4) ^B^	55.6 (5.1–97.6) ^C^	95.8 (15.5–99.4) ^B,C^	0.429	<0.001
SBP (percentile)	52 (6–87) ^B^	62 (2–99) ^C^	91 (5–99) ^B,C^	0.230	<0.001
DBP (percentile)	49 (7–79) ^B^	55 (13–99) ^C^	90 (18–99) ^B,C^	0.201	<0.001
Blood glucose (mg/dL)	95 (84–99) ^B^	96 (79–128) ^C^	107 (73–137) ^B,C^	0.213	<0.001
Blood total cholesterol (mg/dL)	144 (112–169) ^B^	144 (95–199) ^C^	164 (100–237) ^B,C^	0.091	<0.001
NO (μM NO/mM creatinine)	32.2 (10.8–105.5)	37.6 (10.9–108.1)	30.1 (10.3–89.0)	0.146	0.126
Diet					
Fruits & vegetables (servings/day)	3.3 (0.3–13.6)	2.0 (0.0–8.0)	1.7 (0.3–7.6)	0.024	0.063
Whole grains (servings/day)	0.3 (0.0–1.0)	0.3 (0.0–3.0)	0.1 (0.0–1.7)	0.011	0.837
Sodium (mg/day)	2838 (726–5061)	3353 (1884–6528)	3147 (1809–6835)	0.000	0.354
Fish (servings/week)	3.0 (0.0–10.0)	2.0 (0.0–10.0)	2.0 (0.0–16.0)	0.009	0.725
Sugar-sweetened beverages (L/week)	0.8 (0.0–10.2)	0.8 (0.0–8.4)	0.8 (0.0–7.4)	0.011	0.828

Values are expressed as median (range). *p*-values from Krukal Wallis test to refer differences among cardiovascular health score. Effect size from Eta-squared. Significant differences among segments after Bonferroni correction: significant different (*p*-value < 0.05) between: A = Ideal and Intermediate, B = Ideal and Poor; C = Intermediate and Poor. MVPA = Moderate & vigorous physical activity, BMI = body mass index, SBP z-score = z-score of systolic blood pressure, DBP z-score = z-score of diastolic blood pressure. * Dichotomous variable is expressed as a percentage.

**Table 3:** Association between nitric oxide with individual anthropometry, biochemical, physical activity, and dietary parameters.

	Coefficient (β)	Significance (*p*-Value)
Age (month)	0.137	0.100
MVPA (min/day)	0.031	0.711
Anthropometric		
BMI z-score	−0.169	0.029
WC z-score	−0.143	0.134
WHtR z-score	−0.092	0.274
Body fat (%)	−0.196	0.016
Skeletal muscle (%)	0.172	0.036
SBP z-score	−0.263	0.001
DBP z-score	−0.256	0.006
Blood biochemistry		
Glucose (mg/dL)	−0.046	0.581
Triglycerides (mg/dL)	−0.162	0.049
Total cholesterol (mg/dL)	0.121	0.142
HDL-c (mg/dL)	0.167	0.041
LDL-c (mg/dL)	0.853	0.301
Non-HDL-c (mg/dL)	0.037	0.651
Urine analysis		
TPE (mg GAE/g creatinine)	0.250	0.002
Diet		
Fruits/vegetables (servings/day)	0.160	0.050
Whole grains (servings/day)	−0.154	0.061
Sodium (mg/day)	0.168	0.063
Fish (servings/week)	−0.001	0.990
Sugar-sweetened beverages (L/week)	0.108	0.195
CVHs	0.148	0.073

*p*-values and β are of simple linear regression (*n* = 149). MVPA = Moderate & vigorous physical activity, BMI z-score = z-score of body mass index, WC z-score = z-score of waist circumference, WHtR z-score = z-score of waist to height ratio, SBP z-score = z-score of systolic blood pressure, DBP z-score = z-score of diastolic blood pressure, HDL-c = high-density lipoprotein cholesterol, LDL-c = low-density lipoprotein cholesterol, TPE = total polyphenol excretion, GAE= gallic acid equivalent, CVHs = cardiovascular health score. A *p*-value < 0.05 was considered statistically significant.

**Table 4:** Spearman’s correlations of nitric oxide with anthropometry, biochemical, physical activity, and dietary parameters by sex.

	Boys (*n* = 73)		Girls (*n* = 76)	
	Rho	*p*-Value	Rho	*p*-Value
Age (month)	−0.344	0.003	0.102	0.379
MVPA (min/day)	0.005	0.969	0.089	0.447
Anthropometric				
BMI z-score	−0.155	0.192	−0.261	0.023
WC z-score	−0.042	0.724	−0.181	0.117
WHtR z-score	−0.037	0.754	−0.078	0.501
Body fat (%)	−0.095	0.425	−0.232	0.044
Skeletal muscle (%)	0.151	0.202	0.085	0.463
SBP z-score	−0.304	0.009	−0.391	0.001
DBP z-score	−0.227	0.054	−0.317	0.006
Blood biochemistry				
Glucose (mg/dL)	0.064	0.592	−0.206	0.075
Triglycerides (mg/dL)	−0.194	0.101	−0.004	0.973
Total cholesterol (mg/dL)	0.054	0.648	0.353	0.002
HDL-c (mg/dL)	0.123	0.302	0.262	0.022
LDL-c (mg/dL)	0.033	0.779	0.272	0.018
Non-HDL-c (mg/dL)	−0.008	0.946	0.222	0.055
Urine analysis				
TPE (mg GAE/g creatinine)	0.375	0.001	−0.008	0.943
Diet				
Fruits/vegetables (servings/day)	0.132	0.264	−0.147	0.206
Whole grains (servings/day)	−0.051	0.670	−0.349	0.002
Sodium (mg/day)	0.209	0.097	0.028	0.832
Fish (servings/week)	0.043	0.720	−0.091	0.435
Sugar-sweetened beverages (L/week)	−0.030	0.803	−0.296	0.010
CVHs	0.111	0.352	0.217	0.060

MVPA = Moderate & vigorous physical activity, BMI z-score = z-score of body mass index, WC z-score = z-score of waist circumference, WHtR z-score = z-score of waist to height ratio, SBP z-score = z-score of systolic blood pressure, DBP z-score = z-score of diastolic blood pressure, HDL-c = high-density lipoprotein cholesterol, LDL-c = low-density lipoprotein cholesterol, TPE = total polyphenol excretion, GAE= gallic acid equivalent, CVHs = cardiovascular health score. A *p*-value < 0.05 was considered statistically significant.

## Data Availability

The datasets presented in this article are not readily available because there are restrictions on the availability of the data for the SI! Program study, due to signed consent agreements around data sharing, which only allow access to external researcher for studies following project purposes. Requestor wishing to access the database used in this study can make a request to the Steering Committee (SC) chair. Requests to access the datasets should be directed to gsantos@fundacionshe.org, rodrigo.fernandez@cnic.es, juanmiguel.fernandez@cnic.es, and lamuela@ub.edu.

## Supplementary Materials

The following supporting information can be downloaded at: https://www.mdpi.com/article/10.3390/antiox11112140/s1, Figure S1: Process of the different phases of the present study; Figure S2. Flow chart of the population screening process; Table S1. Dietary and information about pathologies or food allergy and intolerance data used for the present study; Table S2. Dietary antioxidant intake by categories of cardiovascular health score; Table S3. Special health situation and pathologies of the participants (self-report).

## Abbreviations

BMI	body mass index
BP	blood pressure
CVH	cardiovascular health
CVHs	cardiovascular health score
DBP	diastolic blood pressure
eNOS	endothelial nitric oxide synthase
NO	nitric oxide
NOX	NADPH oxidases
ROS	reactive oxygen
SBP	systolic blood pressure
TC	total cholesterol
TPE	total polyphenol excretion
