# Supplementary material for: Urinary Nitric Oxide Levels Are Associated with Blood Pressure, Fruit and Vegetable Intake and Total Polyphenol Excretion in Adolescents from the SI! Program
Source: Antioxidants (Basel). 2022 Oct 28;11(11):2140. doi: 10.3390/antiox11112140 (PMC9686949; doi:10.3390/antiox11112140)
Supplement: Supplementary file 1 [file antioxidants-11-02140-s001.zip › antioxidants-1980796-Supplementary.pdf]

Figure S1. Process of the different phases of the present study

Participants of the SI! Program for Secondary Schools at baseline [1]

↓

Data collection

Measurements in fasting:	Physical activity:	Information collected a through self-reported questionnaire:
- Blood pressure<br>- Height<br>- Waist circumference<br>- Bioelectrical impedance:<br>o body weight<br>o body fat percentage<br>o skeletal muscle percentage<br>- Biochemical blood analysis:<br>o glucose<br>o triglycerides<br>o total cholesterol<br>o high density lipoprotein cholesterol<br>o low density lipoprotein cholesterol<br>o non-high density lipoprotein cholesterol<br>- Urine spot sample collection	- The participant used an accelerometer for 7 consecutive days	- Smoking status<br>- Dietetics<br>- Previously diagnosed pathologies

↓

Data processing

- BP z-score
- BMI, BMI z-score, BMI percentile
- WC z-score
- WHtR, WHtR z-score
- Moderate and vigorous physical activity
- Cardiovascular health score

↓

Sample selection
(details in Figure S2)

↓

Urinary biochemical analyses

- Nitric oxide
- Total polyphenol excretion
- Creatinine

↓

Statistical analyses

1. Standardization of variables
2. Determine normality of variables (Kolmogorov–Smirnov test)
3. Identification of differences between sex (T-student), between CVHs categories and NO levels among CVHs (Kruskal–Wallis test with Dunn-Bonferroni correction and eta-squared for effect size) and correlation between NO levels and each study variable (simple linear regression and Spearman)
4. Elimination of collinearity among variables (principal component analysis)
5. Identification of the most important variables associated with NO, fed with the statistically significant variables (forward stepwise selection method)
6. Determination of the influence and global association of study variables with NO (multiple linear regression model)

BP = blood pressure, BMI = body mass index, WC= waist circumference, WHtR = waist to height ratio

Figure S2. Flow chart of the population screening process

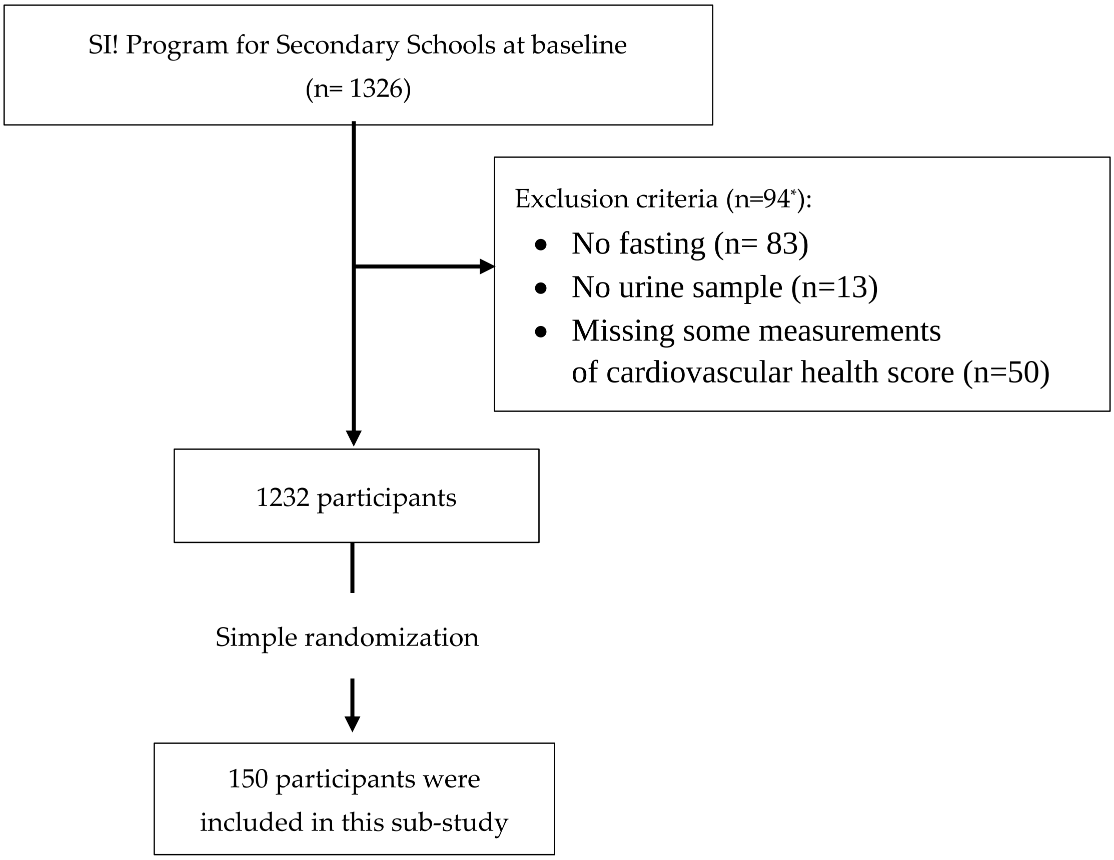

* Some participants have more than one exclusion criteria

Table S1. Dietary and information about pathologies or food allergy and intolerance data used for the present study

**Questions filled by the adolescents**	**Possible answers**
Do you suffer from any of the following diseases?	No / Yes
• Diabetes mellitus type 1	
• Diabetes mellitus type 2	
• Hypertension	
• Hypercholesterolemia	
• Celiac disease	
• Other (specify)	
Do you have any food intolerance?	No / Yes. If yes, to what?
Do you have any food allergy?	
In the last month, how often have you consumed the following foods and drinks?	Select an option*:
• Vegetables, potatoes, or cooked legumes (also combined in the same dish)	• Never/ less than once a week
• Raw vegetables (e.g. mixed in the salad, carrot, cucumber, lettuce, tomato, etc.)	• 1-3 times a week
• Fresh fruits (also blended)	• 4-6 times a week
• Porridge, oat flakes, unsweetened cereals, natural muesli	• Once a day
• Whole-Wheat Bread, Whole-Wheat Rolls, Whole-Wheat Biscuits	• 2 times a day
• Packaged fruit juices	• 3 times a day
• Sweetened beverages including sports drinks, canned or bottled tea, soft drinks, etc.	• 4 or more times a day
• Fresh or frozen fish baked, grilled, steamed	• I do not know
• Fried fish and fish fingers	

Sodium was estimated using the responses of a validated 157-item food frequency questionnaire, which was filled by the family of the participants [2]. * The servings were equated as follows: never/ less than once a week = 0, 1-3 times a week = 2, 4-6 times a week = 5, once a day = 7, 2 times a day = 14, 3 times a day = 21, 4 or more times a day = 30, servings/week respectively, I do not know = no data. Sugar-sweetened beverages were estimated assuming a 0.2 liters/serving, therefore the serving was multiplied by 0.2.

Table S2. Dietary antioxidant intake by categories of cardiovascular health score

	Ideal	Intermediate	Poor	Effect size	p-value
Vitamin A (mcg/day)	873 (209 – 2426)	1145 (416 – 10145)	1184 (532 – 4409)	0.006	0.240
Carotenoids (mcg/day)	3119 (912 – 7881)	4115 (936 – 14649)	3767 (972 – 9152)	0.006	0.231
Retinol (mcg/day)	334 (57 – 1577)	360 (119 – 7948)	387 (80 – 2933)	0.008	0.671
Vitamin E (mg/day)	11.0 (5.0 - 19.9)	12.9 (5.2 – 25.0)	12.7 (5.8 - 29.4)	0.016	0.115
Vitamin C (mg/day)	139 (42 – 516)	210 (52 – 884)	195 (59 - 646)	0.174	0.103

Table S3. Special health situation and pathologies of the participants (self-report).

	Ideal (n=28)	Intermediate (n=77)	Poor (n=44)
Diabetes mellitus	0	0	0
Hypercholesterolemia	0	0	0
Hypertension	0	0	0
Food allergy	0	Egg: 3 participants<br>Fish: 2 participants<br>Kiwi: 1 participant	Squid: 1 participant<br>Kiwi: 1 participant<br>Nuts: 2 participants
Food intolerance	0	Lactose: 1 participant	Didn't mention it: 1 participant
Celiac disease	0	2 participants	0
Other pathologies	0	Esophagitis: 1 participant	Ulcerative colitis: 1 participant

Table show number of participants follow of the pathologies or food allergy or intolerance

References

1. Fernandez-Jimenez, R.; Santos-Beneit, G.; Tresserra-Rimbau, A.; Bodega, P.; de Miguel, M.; de Cos-Gandoy, A.; Rodríguez, C.; Carral, V.; Orrit, X.; Haro, D.; et al. Rationale and Design of the School-Based SI! Program to Face Obesity and Promote Health among Spanish Adolescents: A Cluster-Randomized Controlled Trial. Am. Heart J. 2019, 215, 27–40, doi:10.1016/j.ahj.2019.03.014.
2. Fernández-Ballart, J.D.; Piñol, J.L.; Zazpe, I.; Corella, D.; Carrasco, P.; Toledo, E.; Perez-Bauer, M.; Martínez-González, M.Á.; Salas-Salvadó, J.; Martín-Moreno, J.M. Relative Validity of a Semi-Quantitative Food-Frequency Questionnaire in an Elderly Mediterranean Population of Spain. Br. J. Nutr. 2010, 103, 1808–1816, doi:10.1017/S0007114509993837.
